# “Guide” of muscone modification enhanced brain‐targeting efficacy and anti‐glioma effect of lactoferrin modified DTX liposomes

**DOI:** 10.1002/btm2.10393

**Published:** 2022-08-18

**Authors:** Na Qi, Wenjuan Duan, Duan Gao, Ningzhu Ma, Jianguo Zhang, Jianfang Feng, Aimin Li

**Affiliations:** ^1^ Cancer Center, Integrated Hospital of Traditional Chinese Medicine Southern Medical University Guangzhou China; ^2^ Department of Pharmacy Guilin Medical University Guilin China; ^3^ Department of Pharmacy Affiliated Hospital of Jinggangshan University China; ^4^ Department of Pharmacy Guangxi University of Chinese Medicine Nanning China

**Keywords:** brain targeting, glioma, lactoferrin, modified liposome, muscone

## Abstract

Glioma is one of the most aggressive malignant diseases for human health. It is difficult to resect completely due to their invasiveness. The targeted delivery, as a noninvasive approach, is a major strategy for the development of treatments for brain tumors. Lactoferrin (Lf) receptors are over‐expressed in both brain endothelial cells and glioma cells. Macromolecular Lf modified nanoparticles have been shown to enhance the brain targeting. Muscone is a “guide” drug that have been demonstrated to promote liposomes into the brain by modification. To further enhance the brain‐targeting efficacy of Lf modified carriers, we designed that Lf and muscone dual‐modified liposomes cross blood–brain barrier (BBB) and target to brain for enhanced docetaxel (DTX) brain delivery. The results showed that we successfully prepared Lf and muscone dual‐modified liposomes (Lf‐LP‐Mu‐DTX), the number of Lf molecules connected to the surface of per liposome was 28. Lf‐LP‐Mu‐DTX increased uptake in both U87‐MG cells and hCMEC/D3 cells, enhanced penetration of U87‐MG tumor spheroid and in vitro BBB model, had better in vitro and in vivo anti‐tumor effects. In conclusion, “guide” of muscone modification enhanced brain‐targeting efficacy of Lf modified liposomes, Lf and muscone dual‐modified docetaxel loaded liposomes present a potential brain‐targeting drug delivery system for use in the future treatment of gliomas.

## 1 INTRODUCTION

Gliomas are the most common primary malignant tumors, accounting for about 80% in malignant brain tumors.^1^ Since most gliomas grow invasively in the cerebral hemisphere and cannot be completely removed by the surgery, chemotherapy remains an *important treatment* for malignant *glioma*. However, the presence of blood–brain barrier (BBB) is the biggest obstacle to chemotherapy drugs. BBB strictly controls the delivery of plasma to solutes.^2^ Almost all large‐molecule and over 98% of small‐molecule drugs cannot pass the BBB, and only lipid‐soluble drugs with a molecular mass of less than 600 can cross the BBB via passive diffusion.^3^ Recently, brain targeting delivery systems have represented a promising strategy to improve the treatment of glioma. Brain‐targeted drug *delivery systems* deliver some therapeutic or diagnostic agent to the tumor site in brain, and enhance the accumulation of chemotherapeutic drugs in the tumor site.^4^, ^5^

Recently, natural proteins as drug carriers have advantages including nontoxicity, biodegradability, non‐immunogenic, renewability, and targeting ability.^6^ Protein‐based carriers have been reported in the form of drug conjugates, drug addicts, protein‐mediated/modified nanoparticles, and tissue engineering scaffolds.^6^, ^7^ Lactoferrin (Lf) is a transferrin‐binding cationic glycoprotein of the transferrin family with a molecular weight of 80 kDa.^8^ Lf binds to a specific Lf receptor on the surface of BBB and glioma cells.^9^ Compared to transferrin, Lf with low plasma concentrations can avoid interference with endogenous Lf. Some studies have shown that Lf‐modified delivery systems can promote drugs across the BBB and enhance anti‐glioma effect. The brain‐targeted drug delivery system with Lf as ligand has significantly higher accumulation in the brain than that of Tf and OX26.^10^ Su et al. have successfully developed Lf‐modified NPs as a targeting vector to promote brain tissue uptake of doxorubicin and treat gliomas.^11^ In addition, Song et al. have demonstrated that Lf‐conjugated nanoparticles can deliver drug and imaging probes across endothelial cells to achieve the transport of drugs into brain.^12^

Traditional Chinese Medicines have been practiced in the clinic for hundreds of years. Aromatic resuscitation drugs can guide drugs upward to the brain. Recently, borneol and musk have been developed for enhancing BBB penetration.^13^, ^14^ In addition, borneol could promote co‐administration targeting nanoparticles to enhance the brain targeting efficiency.^15^

Muscone, as the “guide” drug of Traditional Chinese Medicines aromatic resuscitation, with low molecular weight (MW, 238.42) and strong fat‐solubility, can facilitate the permeability of some drugs cross the BBB into the brain.^16^ Further investigation illustrates that the mechanism of muscone promoted the permeability of drugs cross the BBB by inhibiting the expression of P‐glycoprotein (P‐gp) and matrix metalloproteinase‐9 (MMP‐9) and relaxing the tight junctions between epithelial cells.^14^ Recently, aromatic resuscitation drugs are used as ligands to prepare ligand‐modified targeted drug delivery systems by bioconjugate techniques, and to enhance brain targeting effect. A study reported that borneol‐modified PAMAM could increase doxorubicin concentration in the brain and enhance the treatment of glioma.^17^ Another study reported that borneol or muscone were conjugated with bovine serum albumin (BSA) to prepare aromatic resuscitation drugs modified albumin‐based nanoparticles, which can enhance drugs across the BBB for the treatment of glioma.^18^ Our recent research found that muscone modification increased the brain targeting of antibody‐modified liposomes *in vivo*.^4^

To further enhance the brain‐targeted delivery of macromolecules ligands Lf modified carriers, Lf and muscone dual‐modified PEGylated liposome were applied as an active glioma‐targeting drug delivery system. PEGylation prolongs its circulating half‐life and Lf modification enhances its uptake into cancer cells via receptor‐ and adsorption‐mediated transcytosis.^19^ DTX is extracted from cedar paclitaxel needles and then semi‐synthesized. DTX has the better anti‐tumor effect, twice as effective as paclitaxel. DTX has also being reported for the treatment of breast, nonsmall cell lung, prostate cancer, and brain cancers.^5^, ^20^

**SCHEME 1:**
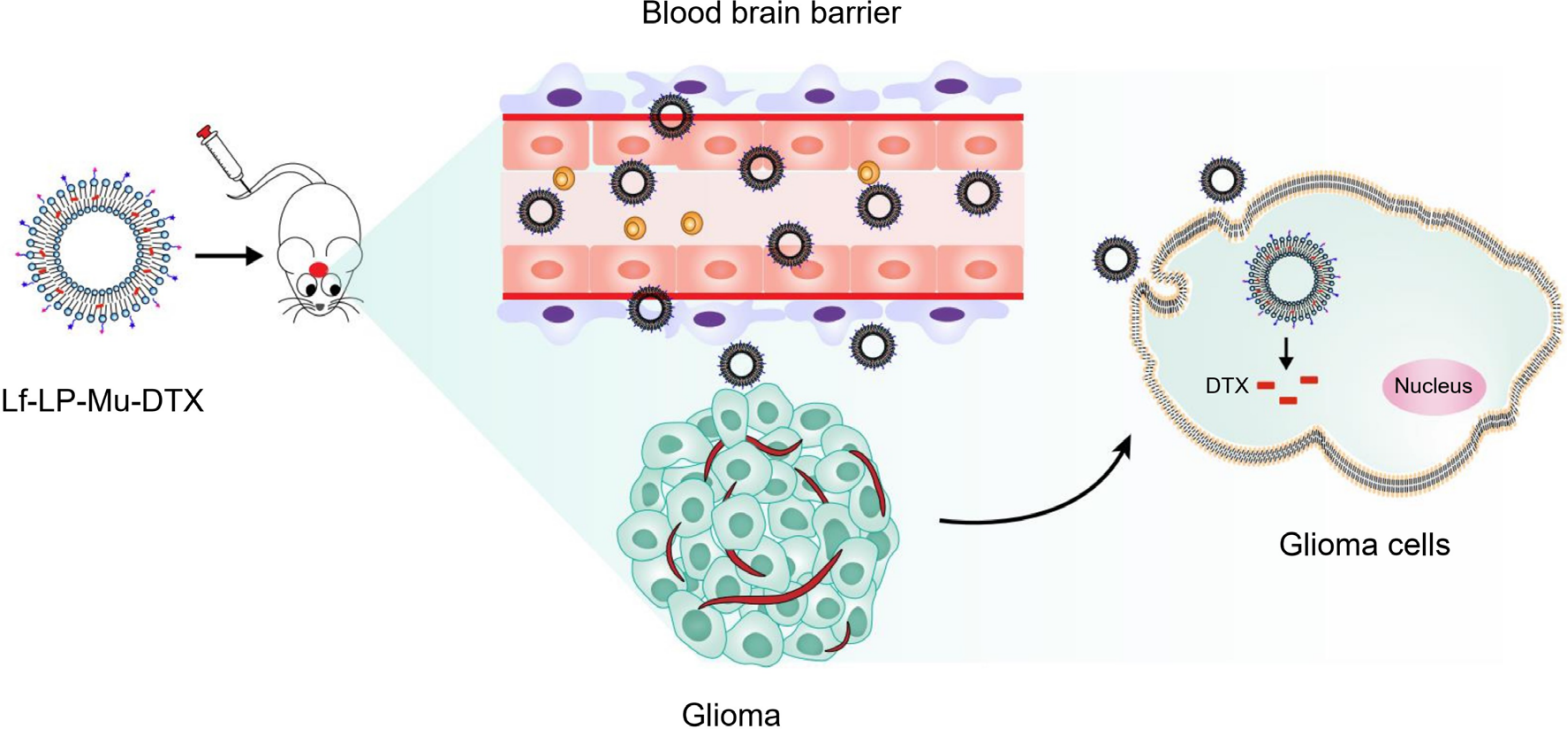
Schematic illustration of Lf‐LP‐Mu‐DTX that crosses the BBB, target the glioma, and exert anti‐glioma effects.

In the present study, we aimed to develop Lf and muscone dual‐modification loaded DTX brain‐targeted delivery systems, to investigate their characterization, and to evaluate their uptake capacity and uptake mechanism, BBB penetration ability and tumor spheroid penetration ability, in vivo brain targeting and therapeutic efficacy of gliomas (Scheme 1).

## 2 MATERIALS AND METHODS

### 2.1 Materials

Egg distearoylphosphatidylcholine (EPC) was purchased from Lipoid GMBH (Germany). DSPE‐PEG and DSPE‐PEG‐maleimide were obtained from Shanghai Ponsure Biotech, Inc. (Shanghai, China). Cholesterol was purchased from Shanghai Xingzhi Chemical Factory (Shanghai, China). DTX was purchased from Wuhan Xinxin Jiali Biological Technology Co., Ltd (Wuhan, China). Coumarin‐6 (C6, purity >98%) was obtained from Sigma Aldrich (St. Louis, MO). 2‐ iminothiolane (Traut's reagent) and Lf were purchased from Thermo (New York, NY). Sephadex G‐50 and Sepharose CL‐4B were purchased from Solarbio Technology Co., Ltd (Beijing, China). 1,1′‐Dioctadecyl‐3,3,3′,3′‐tetramethylindotricarbocyanine iodide (DiR) dye was purchased from Yeasen Biotechnology (Shanghai, China). DMEM medium was obtained from Gibco (CA). Fetal bovine serum (FBS) was purchased from GEMINI (USA). The high‐performance liquid chromatography (HPLC)‐grade solvents used for HPLC were purchased from Xiqiao Chemical Co., Ltd. (Shantou, China). All other chemicals were utilized as analytical grade preparations. DSPE‐PEG_2000_‐Muscone were synthesized as we reported previously.^4^ Ultra centrifugal ultrafiltration tubes (molecular weight cut‐off: 10,000) were from Millipore (Massachusetts, USA).

### 2.2 Cells and animals

U87‐MG glioma cells and immortalized hCMEC/D3 cells were purchased from Shanghai Guandao Biological Engineering Co., Ltd (Shanghai, China). All cells were cultured in DMEM medium with 10% FBS and 1% antibiotics (penicillin and streptomycin). All the cells were cultured at 37°C, with 5% CO_2_.

The male BALB/c nude mice (16–20 g) were obtained from the Shanghai Silaike Laboratory Animal Co, Ltd (Hunan China). All our experiments were performed in compliance with guidelines set by the Guilin Medical University Institutional Animal Care and Use Committee.

### 2.3 Preparation of liposomes

PEG‐LP is consisting of EPC/Chol/DSPE‐PEG_2000_/DTX (100:20:5:3.33, mole ratio). The liposomes containing C6 or DiR were added with fluorescent tracers: C6 (drug‐lipid mole ratio 1: 80) or DiR (drug‐lipid mole ratio 1: 30). Liposomes and Lf modified Liposomes were prepared by thin‐film dispersion method, as our previously described.^4^ To bind Lf, Lf is reacted with Traut's reagent (1:40 molar ratio) in 0.1 mM EDTA/0.1 mM Na‐borate buffer (pH 8.5) to form free thiol group.^21^ After incubation for 120 min at 25°C under N_2_, Lf solution was passed through a Sephadex G‐50 column to remove excess Traut's reagent in phosphate‐buffered saline (PBS, pH 7.4). Thiolated Lf was incubated with liposomes containing DSPE‐PEG_2000_‐MAL for 4 h at 25°C at a molar ratio Lf/DSPE‐PEG_2000_‐MAL of 1:10.^22^ For the containing DSPE‐PEG_2000_‐Muscone groups, the ratio of DSPE‐PEG_2000_‐Muscone to Lf was the same. For the unbound Lf, the LP suspension was passed through a Sepharose 4B‐CL column.^23^ The phospholipid concentrations of liposomes were determined by the Stewart method.^24^ The number of Lf bound to liposomes was tested by Bradford assay.^25^

### 2.4 Physicochemical characterization of liposomes

The average diameter and zeta potential of liposomes were measured by Zetasizer Nano ZS (ZEN3600, Malvern Instruments, UK), as described previously.^26^ All data are repeated in triplicate. The morphology of liposomes was visualized by transmission electron microscopy (TEM; H‐600, Hitachi, Japan) after staining with 1% (w/v) phosphotungstic acid. The concentration of DTX in liposomes was measured via HPLC with UV detection at a wavelength of 222 nm. Chromatographic separation was performed on a Hypersil BDS C18 column (250 mm × 4.6 mm, 5 μm). The mobile phase was composed of a mixture of acetonitrile and water (55:45, v/v). The injection volume was 10 μl, the flow rate was 1.0 ml/min, and total run time 10 min. Encapsulation efficiency (EE) and drug loading capacity (DLC) of DTX in the liposomal samples were determined by dialysis method,^27^ using dialysis bags (MWCO 8000–12,000 Da). Briefly, an aliquot of liposomes suspension was added to the dialysis bag and shake for 8 h in 0.01 M PBS (pH 7.4) to remove free drug. The following equations were used for the calculations:

$$\mathrm{EE}\left(\%\right)=\frac{C_{0}}{C}\times 100\%$$

$$\mathrm{DLC}\left(\%\right)=\frac{C_{0}\times n}{M\times 1000}\times 100\%,$$

where *C* is the total dose of docetaxel (μg/ml), *C*_0_ is the concentration of docetaxel encapsulated in liposomes (μg/ml), *n* is the dilution ratio, and *M* is the total weight of DTX‐LP (mg).

The in vitro release DTX from liposomes was evaluated using the dialysis method as our described previously.^4^ A 2 ml formulation was sealed into a dialysis bag with a molecular weight cut‐off of 8000–12,000 Da, and immersed into 30 ml PBS (pH 7.4) containing sodium salicylate (1 mol/L) at 37°C with a shaking rate of 100 rpm. The release medium in the bag was removed at different time points for up to 72 h, and replaced with the same volume of fresh release medium immediately. The release samples were extracted as our reported previously,^4^ than filtered through 0.22 μm and DTX were determined by HPLC as mentioned above. All experiments were carried out in triplicate.

### 2.5 In vitro hemolysis assay

Blood was taken from the terminal retro‐orbital region of Wistar rats, and fibrinogen was removed using a capillary glass tube. Erythrocytes were washed five times with normal saline and were centrifuged at 1500 rpm for 10 min. Erythrocytes were resuspended in normal saline and were adjusted to 2% concentration (v/v), and 20 μl liposomes, 100 μl 2% erythrocytes dispersion, 100 μl normal saline (negative control), and 1% Triton X‐100 (positive control) were mixed up to EPP tube and incubated at 37°C in a shaking water bath for 3 h, centrifuged at 1500 rpm for 10 min and the supernatant was collected. The samples were determined at 550 nm wave‐length using a spectrophotometer (UV‐2802, Unico, USA). The hemolysis rate was calculated as reported.^28^

Liposomes (20 μl), 100 μl 2% erythrocytes dispersion, 100 μl normal saline (negative control), and 1% Triton X‐100 (positive control) were mixed up to EPP tube and incubated at 37°C in a shaking water bath for 30 min, take 20 μl samples and place it on glass slides, and the erythrocyte agglutination was observed under the microscope.

### 2.6 Cytotoxicity assay

Cytotoxicity of the various liposomes on U87‐MG cells was determined by the MTT assay.^29^ In brief, U87‐MG cells were seeded onto 96‐well plates at a density of 1 × 10^4^ cells/well and incubated at 37°C, 5% CO_2_ for 24 h. Free DTX, PEG‐LP‐DTX, Lf‐LP‐DTX, and Lf‐LP‐Mu‐DTX were diluted into 2, 5, 10, 20, and 40 μg/ml with DMEM and incubation for 24 h. Then, 20 μl MTT solution (5 mg/ml) was added to each well. After incubation for 4 h, the medium containing MTT solution was replaced with 100 μl DMSO to dissolve the formed formazan crystals. Finally, absorbance at 570 nm was measured using a microplate reader (Bio‐Tek, Winooski, VT). Noninoculated cells were used as a negative control.

### 2.7 Cellular uptake

To evaluate the cellular uptake of liposomes, primary cultured U87‐MG cells were seeded into six‐well plates and cultured overnight at 37°C. After applying 5 μg/ml C6 of different C6‐loaded liposomes: PEG‐LP‐C6, Lf‐LP‐C6, Lf‐LP‐Mu‐C6, and Lf‐LP‐C6 + Mu were cultured with U87‐MG cells for 4 h at 37°C.^30^ Cells were trypsinized then collected, and suspended in PBS. The fluorescence intensities of C6 in cells were determined by flow cytometry (FACS AriaIII, BD Biosciences, Franklin Lakes, NJ). The wavelength of C6 emission was 465 nm, and FL2‐H filter (505 nm) was used for the collection of fluorescence intensity. The events collected were 10,000 and the data were analyzed through FCS.

The intracellular localization of C6 in U87‐MG cells was observed with a fluorescence microscope (DM500, Zeiss, Germany). Cells were grown in glass coverslips and treated with 5 μg/ml C6 of different C6‐loaded liposomes: PEG‐LP‐C6, Lf‐LP‐C6, Lf‐LP‐Mu‐C6, and Lf‐LP‐C6 + Mu were cultured with U87‐MG cells at 37°C. After 4 h incubation, cells were fixed with 4% paraformaldehyde for 20 min at room temperature. The cells were washed three times with PBS and incubated with 5 μg/ml Hochest 33342 for 10 min to stain the nuclei.^31^ The cells were washed three times with PBS, and the slides were air‐dried and mounted.

The mechanism of cellular uptake was further evaluated by some endocytic inhibitors. The cells were preincubated with endocytic inhibitors (15 μg/ml amiloride, 5 μg/ml colchicine, 2 μg/ml cytochalasin D, 10 μg/ml chlorpromazine, 5 μg/ml filipin,^32^, ^33^ 1 mg/ml Lf, and 0.4 μg/ml muskone for 30 min at 37°C), compared with that of the noninhibited control, then Lf‐LP‐Mu‐C6 were co‐cultured with cells for 4 h. Quantitative analysis of the intracellular fluorescence intensity was performed by a filter‐type multi‐function microplate reader (Infinite F500, Tecan Austria GmbH, Grodig, Austria).

Relative uptake efficiency(%) = cellular uptake fluorescent intensity of endocytosis inhibitors/cellular uptake fluorescent intensity of blank control × 100%.^33^, ^34^

### 2.8 Tumor spheroids penetration

The three‐dimensional tumor spheroids were prepared as reported previously.^4^ U87‐MG cells were seeded at 5 × 10^3^ cells/well in 96‐well plates pre‐coated with low‐melting agarose (2%, w/v), shaken for 5 min, and incubated for 6 days. When the tumor spheroids reached around 400 μm in *diameter*, and they were incubated with C6‐loaded formulations (PEG‐LP‐C6, Lf‐LP‐C6, Lf‐LP‐Mu‐C6, and Lf‐LP‐C6 + Mu) for 4 h at 37°C. As reported,^4^ the concentration of C6 was 10 μM in C6 loaded formulations, and muscone is 0.4 μg/ml in Lf‐LP‐C6 + Mu. After incubation, the spheroids were washed with cold PBS for three times, and fixed with 4% paraformaldehyde overnight. Then the penetrating capacity of formulations in the tumor spheroids was observed under a confocal fluorescence microscope.

### 2.9 Endothelial permeability test of hCMEC/D3 cells

The model of BBB constructed by hCMEC/D3 cells as reported by Lopalco et al.^35^ Cells were cultured on a 12‐transwell insert, transendothelial electrical resistance (TEER) and the quality of the cell monolayer was measured and compared to previously reported values.^36^ Endothelial permeability of formulations across cell monolayers was carried out according to previously described protocols.^4^

The apparent permeability coefficient (*P*_app_) was calculated according to the following:

*P*_app_ = (*dM*/*dt*)/(*A* × *C*_0_), where *dM*/*dt* is the cumulative measured fluorescence intensity in the receiver chamber per unit time, *A* is the surface area of the insert membrane, and *C*_0_ is the initial concentration of coumarin 6 in the donor chamber.^37^

The transport efficiency was calculated according to the following: The transport efficiency = the cumulative measured fluorescence intensity in the receiver chamber/initial fluorescence intensity in the donor chamber.^38^

All experiments were performed in triplicate.

### 2.10 In vivo imaging

The U87‐MG in situ glioma models were established according to our previous reported.^4^On the 7th day after tumor implantation, three nude mice were randomly selected for magnetic resonance imaging (MRI) observation. The other brain glioma nude mice were randomly divided into four groups (*n* = 3), PEG‐LP‐DiR, Lf‐LP‐DiR, Lf‐LP‐Mu‐DiR, Lf‐LP‐DiR + Mu (gavage), and the mice were injected 200 μl liposomes loaded DiR through the tail vein. After 24 h, the mice were killed and subjected to cardiac perfusion using normal saline and 4% paraformaldehyde, and then the brain, heart, liver, spleen, lung, and kidney were collected for further fluorescent imaging (Ex = 740 nm/Em = 790 nm).

### 2.11 In vivo anti‐glioma effect

We established an orthotopic U87‐MG model in nude mice as above described in vivo imaging. At 6 days after tumor inoculation, nude mice were randomly divided into five groups with 10 mice in each group. The groups were as follows: Model, PEG‐LP‐DTX, Lf‐LP‐DTX, Lf‐LP‐Mu‐DTX, and Lf‐LP‐DTX + Mu (gavage). The mice in the treatment group were given doses equivalent to DTX of 5 mg/kg by intravenous infusions at 6, 9, 12, and 15 days, the survival curve of mice in each group was recorded and the survival curve was drawn.^4^

### 2.12 Statistical analysis

Eight mice were used per group for in vivo anti‐glioma effect of animal study, and three mice were used per group for in vivo imaging of animal study. All experiments were performed in triplicate unless otherwise stated, and the results were presented as mean ± SD. Data were analyzed using the GraphPad Prism software. Differences between the two groups were evaluated by Student's *t*‐test or one‐way ANOVA analysis. Kaplan–Meier survival curves were used to present the differences in the survival of glioma‐bearing mice with different treatments. Differences were considered statistically significant at *p* < 0.05 (*), and very significant at *p* < 0.01 (**).

## 3 RESULTS

### 3.1 Preparation and characterization of LP

**FIGURE 1:**
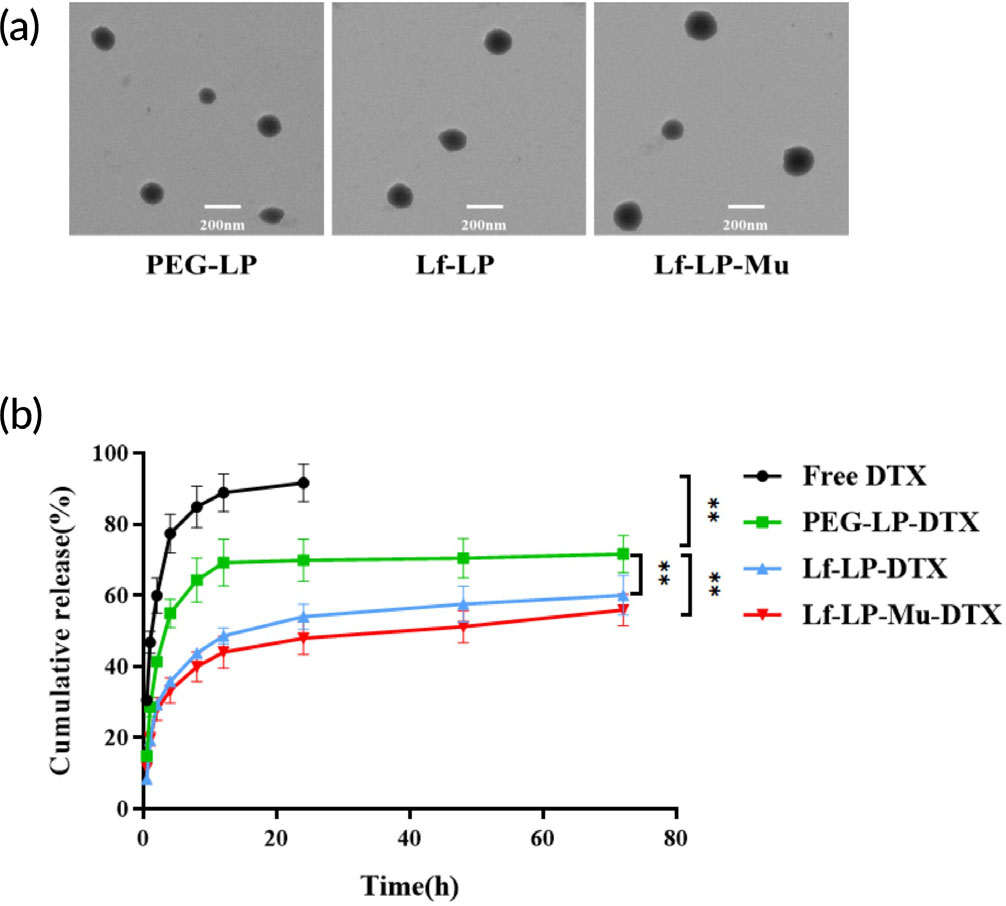
(a) TEM image of DTX‐loaded liposomes. (b) The in vitro release profiles of DTX‐loaded liposomes in PBS (*n* = 3, mean ± SD)

The liposomes with Lf and Muscone modification were prepared by thin‐film dispersion method. The particle sizes and zeta potential of DTX‐loaded liposomes were shown in Table 1. PEG‐LP showed a particle size of 110.0 ± 1.2 nm and a zeta potential of −35.2 ± 1.4 mV. Compared with that of the PEG‐LP, an increase of about 35 nm in particle sizes and a small decrease in zeta potential was observed in Lf‐LP and Lf‐LP‐Mu. The results might be due to macromolecular cationic Lf modified on the surface of liposomes. Polydispersity indexes (PDI) of all liposomes with the requirements of injection (PDI <0.3). The EE and DLC of PEG‐LP, Lf‐LP and Lf‐LP‐Mu were 87.56 ± 5.05%, 79.27 ± 4.99%, 81.68 ± 2.33%, and 2.06 ± 0.06%, 1.67 ± 0.16%, 1.79 ± 0.15%, respectively. The morphology of liposomes was observed by transmission electron microscopy (TEM), shown in Figure 1a, their average diameter is 100–150 nm, which was in an agreement with sizing by dynamic laser scattering. The content of Lf on the surface of liposomes was determined by the Bradford method. The amount of Lf coupled with liposomes was calculated according to the reports that a liposome with 100 nm particle size contains ∼100,000 molecules of phospholipids.^39^ On experimental conditions (molar ratio of DSPE‐PEG_2000_‐maleimide to Lf 1:10), average number of Lf calculated on the surface of each liposome (Lf‐LP or Lf‐LP‐Mu) was around 28.

**TABLE 1:** Particle size and Zeta potential of DTX‐loaded liposomes (Mean ± SD, *n* = 3)

Liposomes	Particle size(nm)	PDI	Zeta potential (mV)	EE	DLC
PEG‐LP	110.0 ± 1.2	0.192 ± 0.002	−35.2 ± 1.4	87.56 ± 5.05%	2.06 ± 0.06%
Lf‐LP	146.2 ± 0.6	0.222 ± 0.017	−27.9 ± 1.1	79.27 ± 4.99%	1.67 ± 0.16%
Lf‐LP‐Mu	144.6 ± 3.1	0.198 ± 0.014	−30.3 ± 1.6	81.68 ± 2.33%	1.79 ± 0.15%

The in vitro drug release of formulations was evaluated in 0.1 M PBS (pH 7.4), the results are shown in Figure 1b. As it can be seen, about 90% DTX was released from the DTX solution within 24 h, PEG‐LP‐DTX showed sustained release property with a release rate of 70% at 72 h. Lf‐LP‐DTX exhibited slightly slower release properties than PEG‐LP‐DTX, the release curve from Lf‐LP‐Mu‐DTX exhibited more slower sustained DTX release properties than Lf‐LP‐DTX.

### 3.2 In vitro hemolysis studies

**FIGURE 2:**
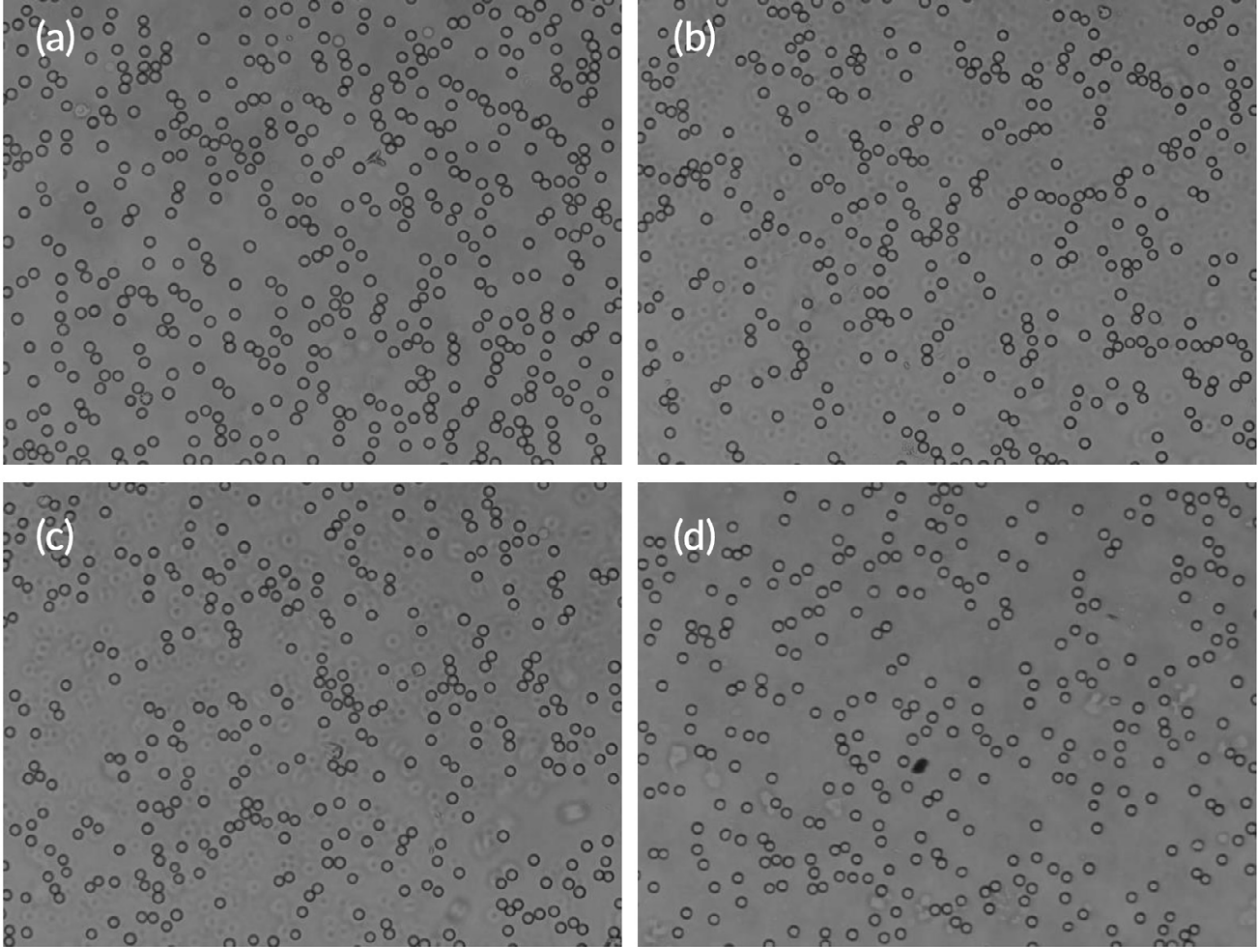
Images of erythrocytes aggregation treated with different liposomes: (a) normal saline, (b) PEG‐LP, (c) Lf‐LP, and (d) Lf‐LP‐Mu

Erythrocytes hemolysis assay, and erythrocyte agglutination of formulations was performed for evaluating the in vitro safety. Erythrocytes hemolytic rates in PEG‐LP, Lf‐LP, and Lf‐LP‐M groups were 4.78%, 4.85%, and 4.59%, which are in the acceptable range for human body. The results were consistent with about 5% hemolysis rates of traditional liposomes.^40^ After different liposomes were incubated with erythrocytes, the morphology of erythrocytes in each group was shown in Figure 2. Compared with the normal saline group, PEG‐LP, Lf‐LP, and Lf‐LP‐M groups did not cause erythrocyte agglutination and erythrocyte abnormity. In general, the result showed that modified liposomes exhibited negligible toxicities on erythrocyte hemolysis assay and erythrocyte agglutination.

### 3.3 Cytotoxicity assay

**FIGURE 3:**
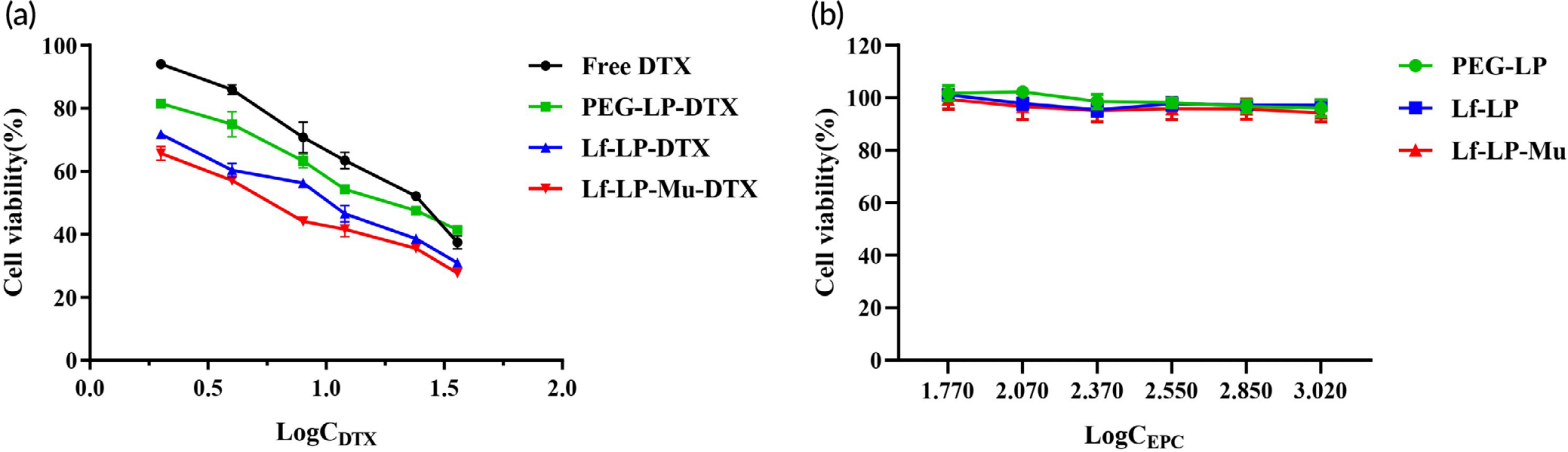
In vitro cytotoxicity of DTX‐loaded liposomes in U87‐MG cells. (a) DTX‐loaded liposomes (C_DTX_, μg/ml), (b) Blank liposomes (C_EPC_, μg/ml). Data represented the mean ± SD (*n* = 3)

In vitro cytotoxicity of free DTX and different DTX‐loaded liposomes on U87‐MG cells was measured by MTT assay. As shown in Figure 3a, after 24 h incubation with free DTX and formulations, all groups loaded with DTX concentration from 2 to 40 μg/ml showed concentration‐dependent cytotoxic effects on U87‐MG cells. According to the results of cytotoxicity, IC50 values of DTX, PEG‐LP‐DTX, Lf‐LP‐DTX, and Lf‐LP‐Mu‐DTX were 22.62, 19.43, 9.96, and 6.42 μg/ml, respectively. Compared with free DTX and PEG‐LP‐DTX, Lf‐LP‐DTX group exhibited higher cytotoxicity on U87‐MG cells. When DTX was loaded into liposomes, which could be attributed to higher intracellular delivery of DTX from enhancing cell uptake,^41^ and leads to higher cytotoxicity. The results indicated that LF‐modified liposomes enhanced cytotoxicity which was related to increased cellular uptake by receptor‐ and adsorption‐mediated endocytosis pathway on the surface of glioma cells.^11^, ^19^ Moreover, Lf‐LP‐Mu‐DTX group showed the highest in vitro antitumor activity than other groups, which might be due to the combined action of LF modification increasing the targeting recognition of LfR and muscone modification enhancing internalization of glioma cells. After 24 h treatment, no significant cytotoxicity was found for blank liposomes (Figure 3b).

### 3.4 In vitro cellular uptake

**FIGURE 4:**
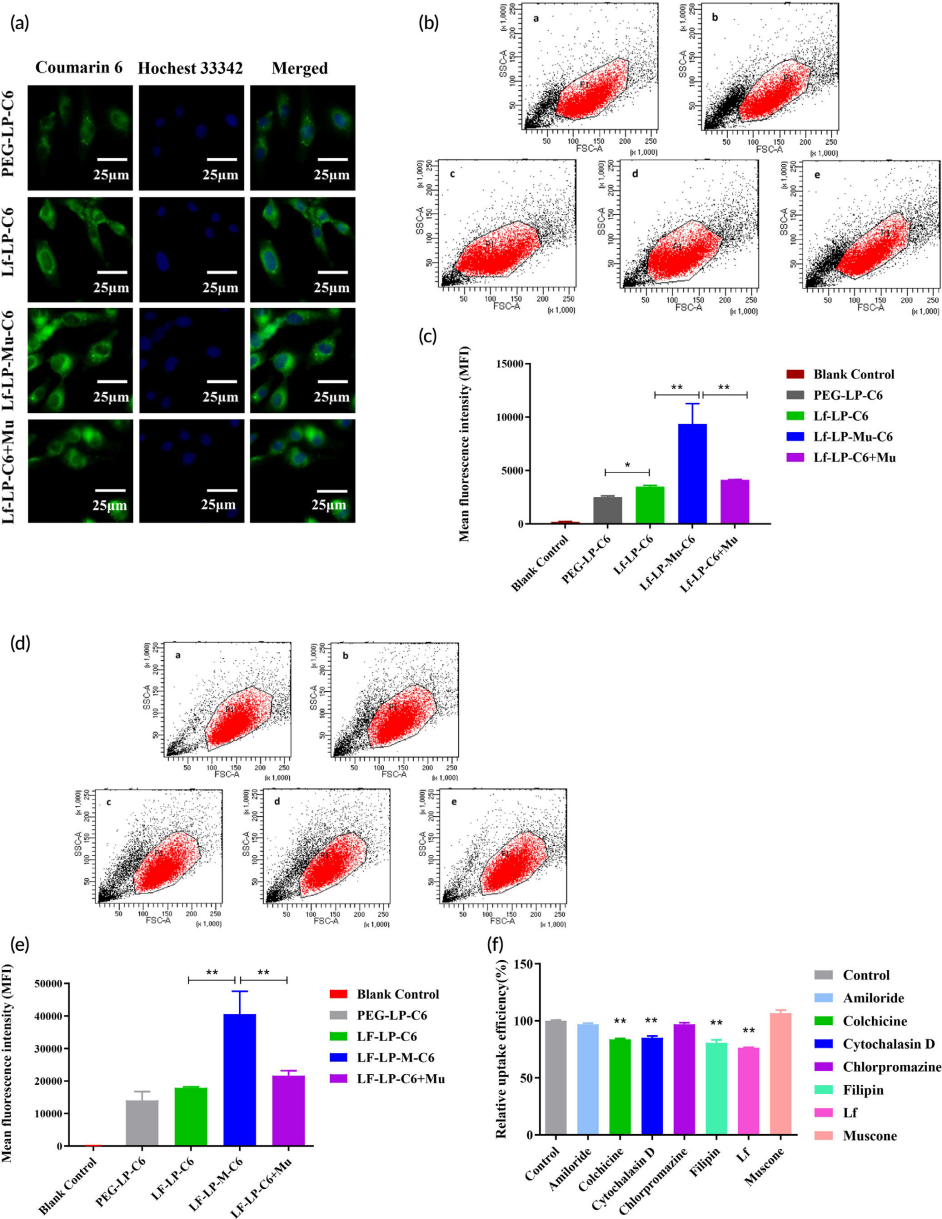
Cellular uptake of C6‐loaded formulations at 4 h. (a) Cellular uptake of C6‐loaded formulations (green staining) incubated with U87‐MG cells for 4 h, and the nuclei were stained by Hochest33342 (blue staining). (b) Flow cytometry analysis of U87‐MG cells after treated with C6‐loaded formulations. A: Blank control, B: PEG‐LP‐C6, C: Lf‐LP‐C6, D: Lf‐LP‐Mu‐C6, and E: Lf‐LP‐C6 + Mu. (c) The mean fluorescence intensity values of C6‐loaded formulations in U87‐MG cells were semi‐quantitatively analyzed according to the results of flow cytometry analysis. (d) Flow cytometry analysis of hCMEC/D3 cells after treated with different C6 loaded formulations. A: Blank control, B: PEG‐LP‐C6, C: Lf‐LP‐C6, D: Lf‐LP‐Mu‐C6, E: Lf‐LP‐C6 + Mu. (e) The mean fluorescence intensity values of C6 loaded formulations in hCMEC/D3 cells were semi‐quantitatively analyzed according to the results of flow cytometry analysis. (f) Uptake mechanism of Lf‐LP‐Mu‐C6 in U87‐MG cells

Cellular uptake of C6‐loaded PEG‐LP, Lf‐LP, and Lf‐LP‐Mu in U87‐MG cells were qualitatively and quantitatively determined by fluorescence microscopy and flow cytometry, respectively. The cellular uptake of formulations was determined by fluorescence microscopy imaging (Figure 4a). In Figure 4b,c, the mean fluorescence intensity of Lf‐LP‐C6, Lf‐LP‐Mu‐C6, and Lf‐LP + Mu‐C6 in U87‐MG cells was 1.4, 3.8, and 1.7 times than PEG‐LP‐C6 by quantitative analysis of flow cytometry. The results showed that the cellular uptake of Lf‐LP‐C6 in U87‐MG cells was significantly higher than that of PEG‐LP‐C6 (*p* < 0.05), and the cellular uptake of Lf‐LP‐Mu‐C6 in U87‐MG cells was significantly higher than that of Lf‐LP‐C6 (*p* < 0.01) and Lf‐LP + Mu‐C6 (*p* < 0.01). However, compared with the Lf‐LP‐C6 group, muscone co‐incubation with Lf‐LP‐C6 group (Lf‐LP + Mu‐C6) did not significantly enhanced cellular uptake of U87‐MG cells (*p* > 0.05).

Figure 4d,e shows the quantitative uptake of C6‐loaded formulations after incubation with hCMEC/D3 cells for 4 h. The mean fluorescence intensity of Lf‐LP‐C6, Lf‐LP‐Mu‐C6, and Lf‐LP + Mu‐C6 in hCMEC/D3 cells was 1.2, 2.9, and 1.5 times than PEG‐LP‐C6. Lf‐LP‐Mu‐C6 remarkably enhanced the mean fluorescence intensity in hCMEC/D3 cells compared with Lf‐LP‐C6 (*p* < 0.01) and Lf‐LP‐C6 + Mu (*p* < 0.01), and Lf‐LP + Mu‐C6 group did not show significantly higher cellular uptake in hCMEC/D3 cells than Lf‐LP‐C6 group (*p* > 0.05), which is in conformity to the results of U87‐MG cells. However, the cellular uptake of Lf‐LP‐C6 in hCMEC/D3 cells was not significantly higher than that of PEG‐LP‐C6 (*p* > 0.05), the possible reason is that a limited number of receptors on the surface of hCMEC/D3 cells leads to no obvious increasing on cellular uptake of Lf‐LP‐C6. In addition, after treatment of formulations, number of positive cells, positive cells rate and the corresponding figure on U87‐MG cells and hCMEC/D3 cells were measured by flow cytometry showed in Tables S1 and S2

### 3.5 Mechanism of cellular uptake

In order to identify the cellular uptake mechanisms of liposomes, U87‐MG cells were pretreated with some endocytic inhibitors,^32^, ^33^, ^42^ including amiloride (macoropinocytosis inhibitor), colchicine (a disrupting agent of microtubule required for macropinocytosis), cytochalasin D (actin‐disrupting agent), chlorpromazine (clathrin‐mediated endocytosis inhibitor), and filipin (caveolin inhibitor), free Lf and muskone, respectively. Then the cellular uptake of Lf‐LP‐Mu‐C6 was measured. From Figure 4f, compared with control group (no inhibitor), the cellular uptake of Lf‐LP‐Mu‐C6 decreased significantly after treatment with colchicine (*p* < 0.01), cytochalasin D (*p* < 0.01), filipin (*p* < 0.01), and free Lf (*p* < 0.01).

### 3.6 Tumor spheroid penetration

**FIGURE 5:**
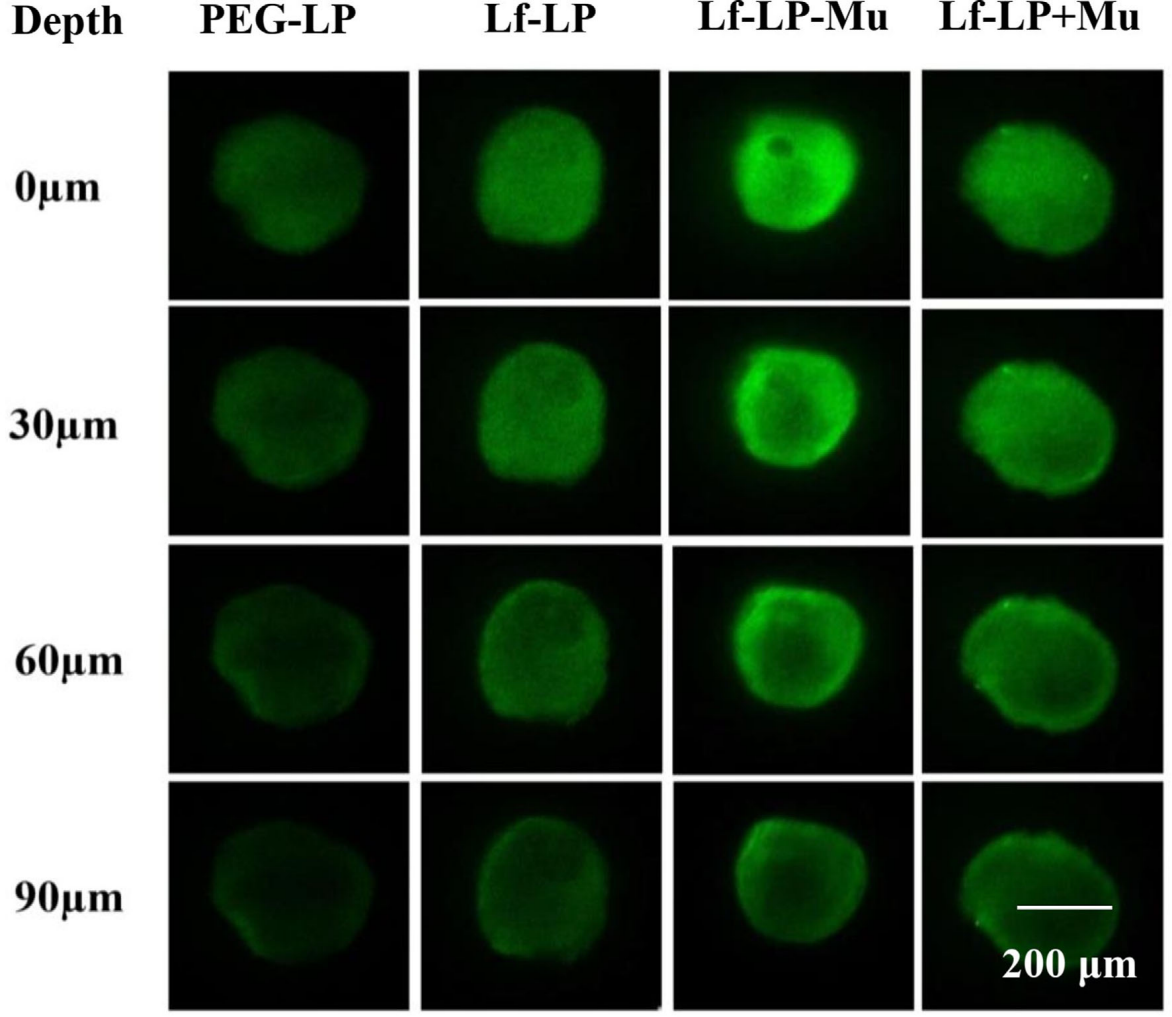
Tumor spheroids penetration of C6 loaded liposomes under a CLSM from the top to middle layers of U87‐MG tumor spheroids for 4 h

In order to evaluate in vitro solid tumor spheroids penetration of the formulations, the 3D U87‐MG tumor spheroids were analyzed under a confocal laser scanning microscope (CLSM). U87‐MG tumor spheroids were incubated with the C6‐loaded formulations, and fluorescence images were analyzed under a CLSM from the top to middle layers of the tumor spheroids. The results are shown in Figure 5. The fluorescence intensity of different C6‐loaded formulations penetrating into the core of the tumor spheroids was in the following order: Lf‐LP‐Mu‐C6 > Lf‐LP + Mu‐C6 > Lf‐LP‐C6 > PEG‐LP‐C6.

### 3.7 Endothelial permeability of hCMEC/D3 cells

**FIGURE 6:**
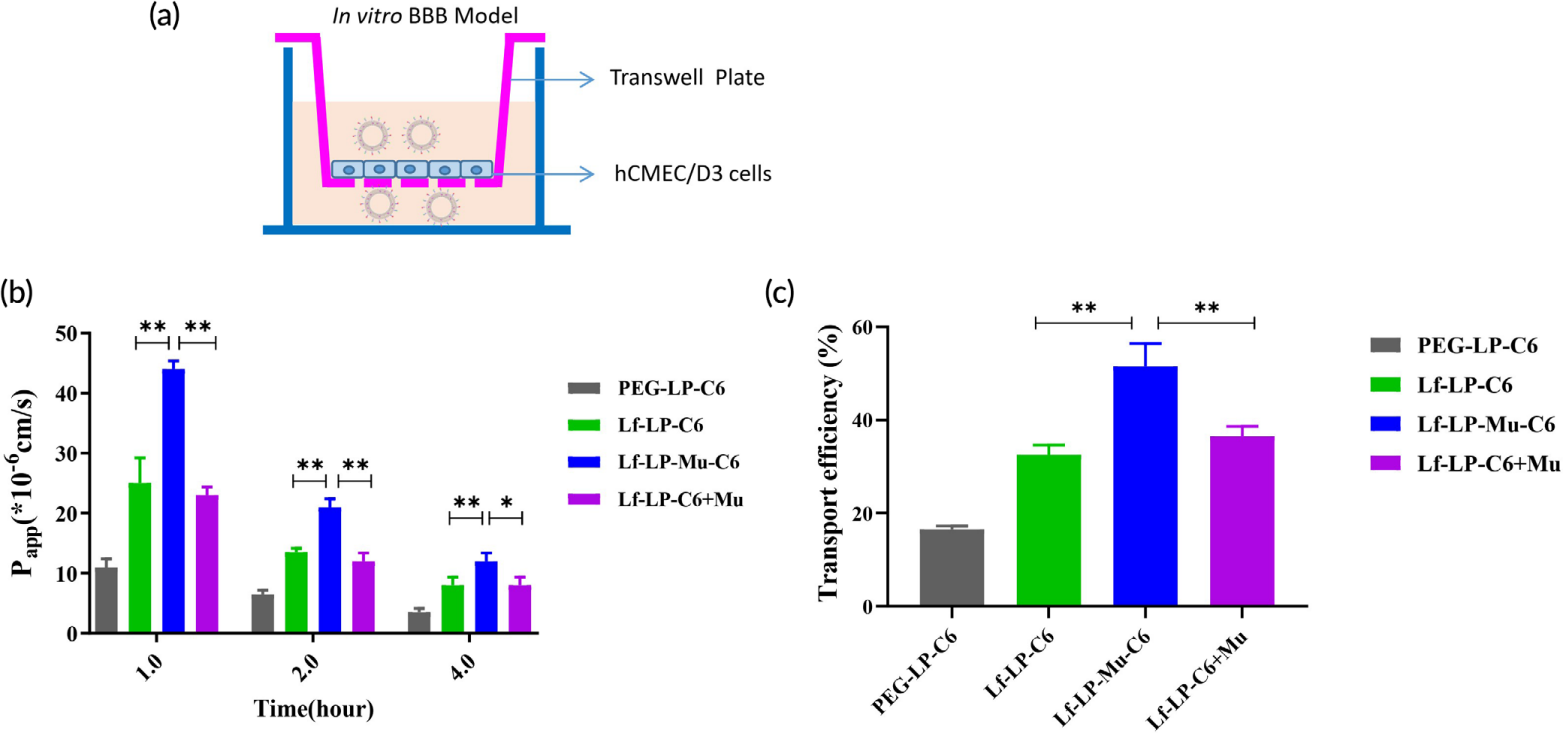
(a) Scheme of the Transwell model. (b) The *P*_app_ of C6 loaded liposomes through hCMEC/D3 cell monolayers at 1, 2, and 4 h. (c) The transport efficiency of C6 loaded liposomes through hCMEC/D3 cell monolayers

Here, we evaluated in vitro transportation of liposomes across the BBB using hCMEC/D3 cell monolayers cultured on Transwell inserts (Figure 6a). At 12 days, the TEER value of cell monolayers was between 65 and 89 Ω·cm^2^ as reported,^35^, ^43^ indicating the cell monolayers were suitable for further transport study. The results of *P*_app_ in Figure 6b showed that Lf‐LP‐C6 provide higher *P*_app_ across the monolayer compared to PEG‐LP‐C6, and Lf‐LP‐Mu‐C6 showed higher *P*_app_ than Lf‐LP‐C6 and Lf‐LP‐C6 + Mu at each time point. With the extension of time, *P*_app_ of all formulations decreased, because the concentration of liposomes in Transwell inserts chamber decreased gradually.

The transport efficiency is shown in Figure 6c. The transport efficiency of PEG‐LP‐C6 is 16.1%, Lf‐LP‐C6 showed a transport efficiency of 32.4%, whereas Lf‐LP‐Mu‐C6 had the highest transport efficiency of 51.0%, 1.6‐fold higher than that of Lf‐LP‐C6, and 1.4‐fold higher than that of Lf‐LP‐C6 + Mu. Lf and muscone dual‐modified liposomes significantly increased the transport efficiency of in vitro BBB model than that of Lf‐LP‐C6 (*p* < 0.01) and Lf‐LP‐C6 + Mu (*p* < 0.01). The results were consistent with the cellular uptake by hCMEC/D3 cells.

### 3.8 In vivo imaging

**FIGURE 7:**
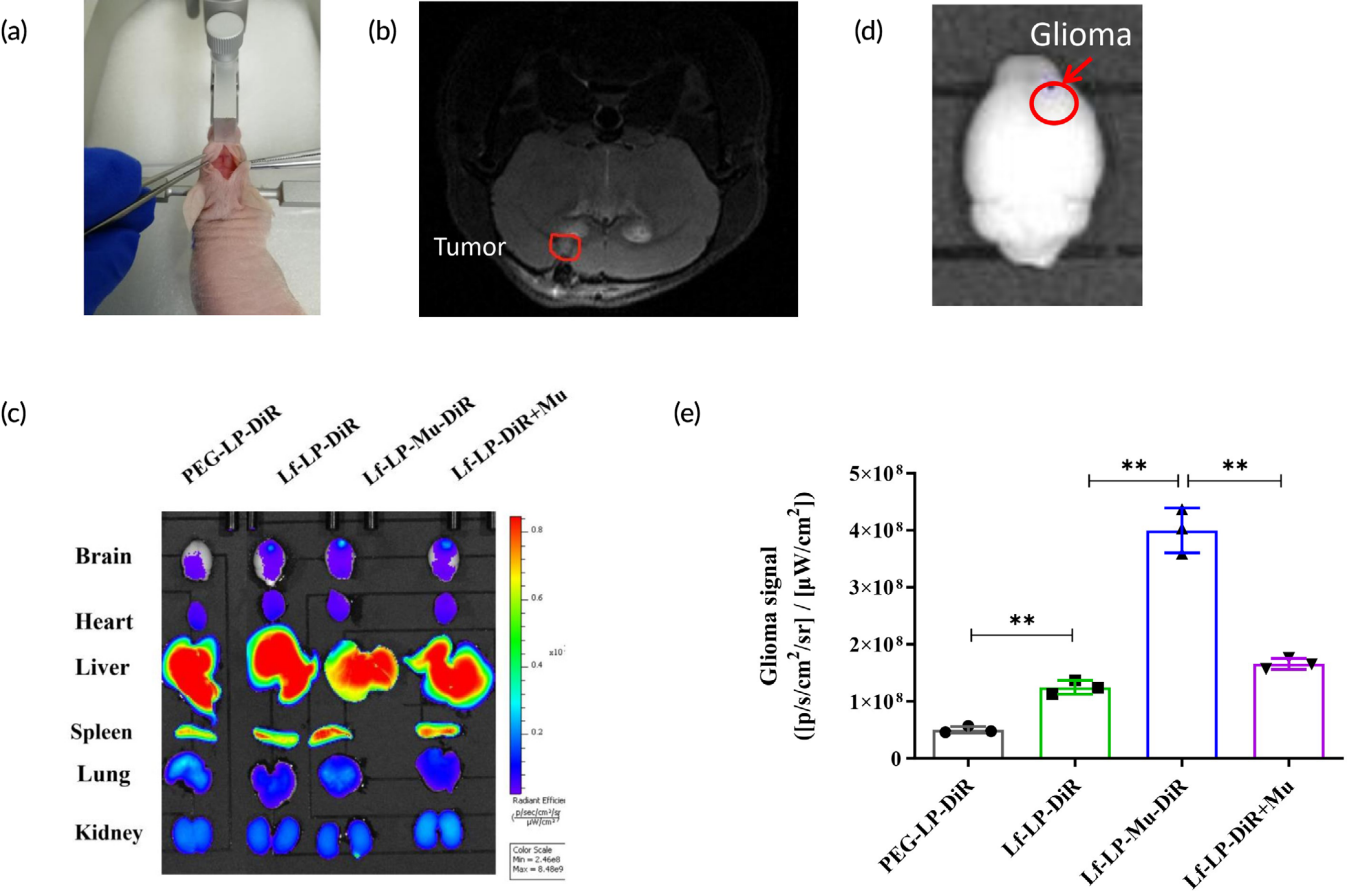
(a) The implantation photo of intracranial U87‐MG glioma. (b) The brain Magnetic resonance imaging (MRI) image of glioma‐bearing nude mice. (c) Ex vivo brain images of nude mice after intravenous administration of DiR‐loaded liposomes for 24 h. (d) Quantitative region of fluorescence signal in glioma. (e) The mean fluorescence intensity values of DiR‐loaded liposomes at the orthotopic glioma site were semi‐quantitatively analyzed (*n* = 3)

To confirm the in vivo targeting ability of liposomes, the in vivo distribution of DiR‐loaded liposomes in the glioma‐bearing nude mice was performed by the fluorescent imaging system. The implantation photo of intracranial U87‐MG glioma is displayed in Figure 7a, brain magnetic resonance imaging (MRI) image of glioma‐bearing nude mice is shown in Figure 7b, which had confirmed brain tumor formation.

After 24 h, the brain, heart, liver, spleen, lung, and kidney were collected for ex vivo imaging in Figure 7c. In Figure 7c, compared with the PEG‐LP‐DiR group, Lf‐LP‐DiR has been shown to enhance the delivery of the drug into the brain. The accumulation of the Lf‐LP‐Mu‐DiR group in the brain tumor site was much higher than that in Lf‐LP‐DiR and Lf‐LP‐DiR + Mu groups. According to fluorescence imaging of the brain in Figure 7c, the fluorescence signal of the orthotopic glioma in different formulation groups was further semi‐quantitatively analyzed in Figure 7e, the region where we quantified the fluorescence signal of glioma was shown in Figure 7d. In Figure 7e, the fluorescence intensity of Lf‐LP‐DiR group is higher than that of PEG‐LP‐DiR group (*p* < 0.01), and the fluorescence intensity of Lf‐LP‐Mu‐DiR group are higher than that of Lf‐LP‐DiR (*p* < 0.01) and Lf‐LP‐DiR + Mu group (*p* < 0.01), respectively. This is in agreement with the results of cellular uptake and tumor spheroid penetration. For Lf‐LP‐DiR + Mu group, results show that the fluorescence intensity of Lf‐LP‐DiR + Mu group are significantly lower than that of Lf‐LP‐Mu‐DiR group (*p* < 0.01) and somewhat higher than that of Lf‐LP‐DiR group.

### 3.9 In vivo anti‐glioma effect

**FIGURE 8:**
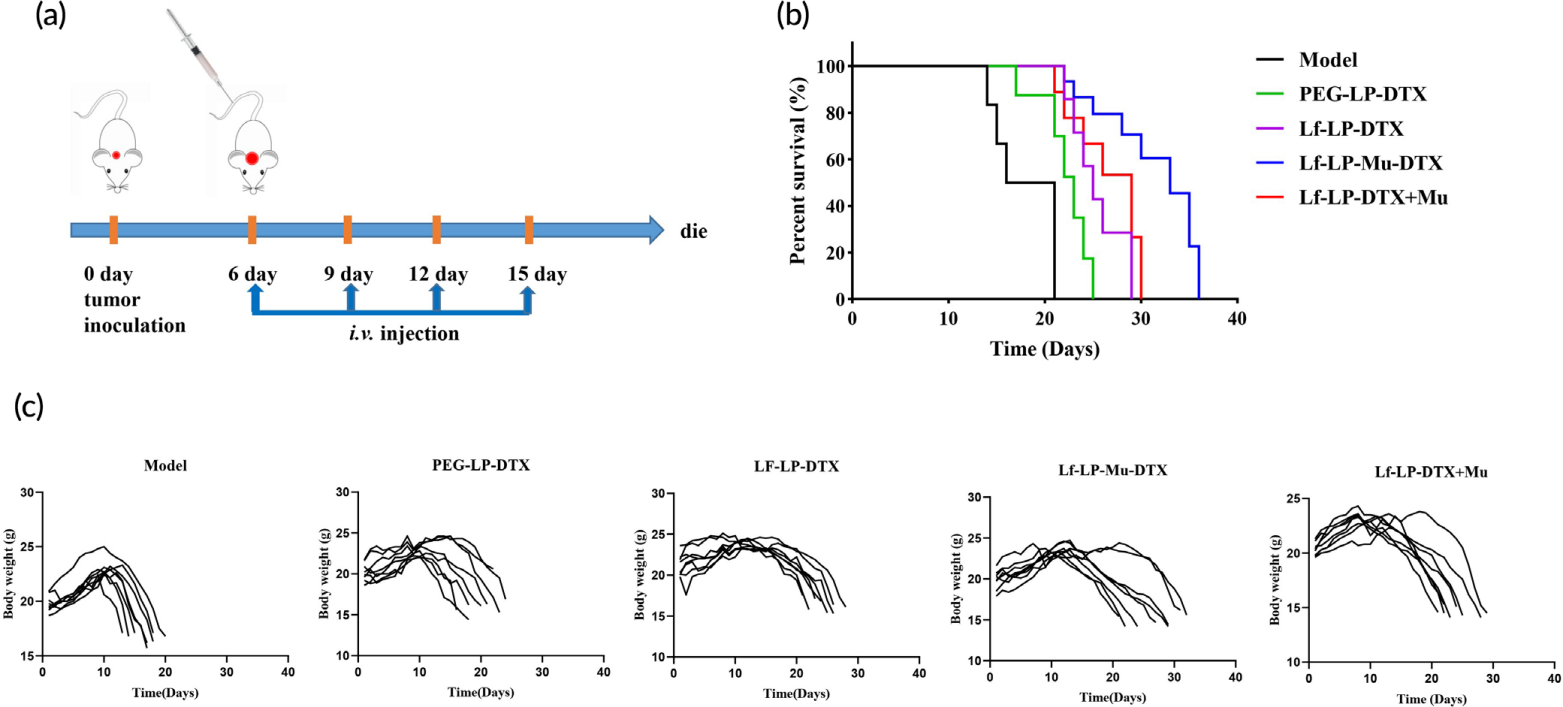
(a) Tumor inoculation and administration routes scheme. (b) Survival curves of glioma‐bearing mice treated with different formulations (*n* = 8). (c) Changes in body weights of individual U87‐MG glioma‐bearing nude mice in each group (*n* = 8)

We use the orthotopic U87‐MG gliomas‐bearing mice to evaluate the anti‐glioma effects of liposomes by investigating the medium survival time. Six days after tumor inoculation, liposomes injection via tail vein was performed every 3 days four times (Figure 8a). From Figure 8b, the median survival time of the LF‐LP‐Mu‐DTX group, Lf‐LP‐DTX + Mu groups, Lf‐LP‐DTX groups, PEG‐LP‐DTX groups was 33, 29, 28, and 22 days, was 1.65×, 1.45×, 1.4×, and 1.1× longer than that of Model group. The results showed that the Lf‐LP‐Mu‐DTX group exhibited the best tumor suppressive effect, which was in agreement with the results of ex vivo imaging. In addition, there was no notable body weight change in all test groups during the treatment course (Figure 8c).

## 4 DISCUSSION

Gliomas are extremely aggressive brain tumors that are difficult to completely surgically removed and have a poor prognosis. Chemotherapy is still an indispensable treatment for glioma. However, the presence of BBB limits the effective delivery of chemotherapeutic drugs. Recently, the functionalization of nanoparticles was demonstrated to be a promising approach for overcoming BBB in brain delivery of anticancer drugs.^33^, ^44^ Some studies reported that Lf was used for a brain‐targeting ligand and enhanced the penetration of the BBB.^45^, ^46^ However, LF alone as a targeting ligand, its targeting efficiency is limited. Recently, the dual targeting delivery systems are developed to promote penetration of the BBB thus improving anti‐tumor effect in vivo.^47^

In the present study, we investigated on promoting BBB penetration and anti‐glioma effect of Lf and muscone dual‐modified DTX liposomes (Lf‐LP‐Mu‐DTX) in vitro and in vivo. Lf and muscone dual modified liposomes with 100–150 nm particle size were obtained and exhibited negligible toxicities on erythrocyte hemolysis assay and erythrocyte agglutination. These characteristics show that it is suitable for system injection. The results of in vitro drug release are shown that Lf‐LP‐DTX exhibited slightly slower release properties than PEG‐LP‐DTX, because the peptide sequences of LF contain many hydrophobic bonds,^48^ and DTX is also hydrophobic, the hydrophobic bonds interaction between LF on the surface of liposomes and DTX that can hinder DTX release from the liposomes. The release profile of Lf‐LP‐M‐DTX exhibited a slower sustained release of DTX than Lf‐LP‐DTX, which might be due to the hydrophobic bonds interaction among muscone, LF, and DTX that may further hinder DTX release. Macromolecular Lf and muscone dual‐modified liposomes can slow DTX release, which makes dual‐modified liposomes have the potential efficacy of delivering high‐dose drug to the tumor cells and enhancing anti‐glioma effect.

The cellular uptake of different C6 loaded formulations in U87‐MG cells was in the following order: Lf‐LP‐Mu‐C6 > Lf‐LP + Mu‐C6 > Lf‐LP‐C6 > PEG‐LP‐C6. The results showed that the cellular uptake of Lf‐LP‐C6 in U87‐MG cells was significantly higher than that of PEG‐LP‐C6 (*p* < 0.05), which indicated that Lf‐modified liposomes enhanced the cellular uptake of formulations through recognizing LfR on the surface of glioma cells by a receptor‐mediated endocytosis pathway.^11^, ^49^ And the cellular uptake of Lf‐LP‐Mu‐C6 in U87‐MG cells was significantly higher than that of Lf‐LP‐C6 (*p* < 0.01) and Lf‐LP + Mu‐C6 (*p* < 0.01). The fluorescence intensity of glioma cells was further enhanced by Lf and muscone dual‐modification, and Lf and muscone dual‐modification have a synergistic effect on cellular uptake. We speculated that muscone modification increased the lipophilicity of the targeted delivery system, which enhanced cellular uptake of Lf‐LP‐Mu‐C6, consistent with literature reported.^18^ Yet, muscone co‐incubation has little effect on cellular uptake of the targeted delivery system. In addition, cationic Lf modified on the surface of liposomes could easily absorb onto the surface of the negative tumor cell membrane via electrostatic interaction, and increase uptaking by adsorption‐mediated endocytosis pathways.^19^ Some studies reported that LfR‐mediated endocytosis was unidirectional, which might increase the uptake of Lf‐modified liposomes.^11^, ^50^

In the mechanism study of cellular uptake, from Figure 4f, compared with control group (no inhibitor), the cellular uptake of Lf‐LP‐Mu‐C6 decreased significantly after treatment with colchicine (*p* < 0.01), cytochalasin D (*p* < 0.01), filipin (*p* < 0.01). The result suggested that the internalization of Lf and muscone dual‐modified liposomes in U87‐MG cells involved in the way of microtubule‐mediated macropinocytosis, actin, and caveolin‐mediated endocytosis pathways.^32^, ^42^ And then, our results showed that free LF significantly reduced the cellular uptake of Lf‐LP‐Mu‐C6 in U87 cells compared with control group (*p* < 0.01), which suggested the internalization of Lf‐LP‐Mu‐C6 involved Lf receptor‐mediated endocytosis, consistent with literature reported.^45^ However, the results showed that muskone did not inhibit cellular uptake, but it can slightly increase cellular uptake. Therefore, we think that muskone does not have a specific blocking effect on endocytosis.

In the study of in vitro cytotoxicity, Lf‐LP‐Mu‐DTX group showed the highest in vitro antitumor activity than other groups, which possibly was due to cellular uptake increasing of the formulations. In addition, the result of tumor spheroid penetration was consistent with that of cellular uptake. LF‐LP‐Mu‐C6 increased penetration into tumor spheroids, which possibly was due to LF modification facilitating cellular internalization of liposomes and muscone modification increasing the permeability of liposomes. LF‐LP‐Mu‐C6 enhancing the penetration ability in tumor spheres was associated with functional targeting liposomes increasing cellular uptake, consistent with literature reported.^51^

During the penetration process of cell monolayers, Lf and muscone dual‐modified liposomes significantly increased transport efficiency of in vitro BBB model than that of Lf‐LP‐C6 (*p* < 0.01) and Lf‐LP‐C6 + Mu (*p* < 0.01). The results were consistent with the cellular uptake by hCMEC/D3 cells. Due to the presence of LfR on the surface of hCMEC/D3 cells, the findings also inferred that Lf‐modified liposomes increased the transport efficiency of hCMEC/D3 cell monolayers by specifically recognizing LfR through a receptor‐ and adsorption‐mediated endocytosis pathway,^19^, ^49^ and muscone modification improved transport efficiency of Lf modified liposomes. The results were consistent with our previous report.^4^

In ex vivo imaging from Figure 7e, the fluorescence intensity of Lf‐LP‐DiR group was higher than that of PEG‐LP‐DiR group (*p* < 0.01), and the fluorescence intensity of Lf‐LP‐Mu‐DiR group was higher than that of Lf‐LP‐DiR (*p* < 0.01) and Lf‐LP‐DiR + Mu group (*p* < 0.01), respectively. The results were in agreement with that of cellular uptake and tumor spheroid penetration. The results showed that Lf‐LP‐Mu‐DiR could effectively penetrate across the BBB and blood–brain tumor barrier (BBTB) and then accumulated at glioma site, which were attributed to the Lf targeted recognition effect of Lf‐LP‐Mu‐DiR with LfR on the surface of BBB and gliomas, and muscone modification to promote the BBB permeability, which may be speculated that modified muscone as hydrophobic unit in Lf and muscone dual‐modified liposomes improve the fluidity of phospholipid molecules in the cell membrane,^52^ increase penetration of modified liposomes into deep tumor tissue. For Lf‐LP‐DiR + Mu group, results showed that the fluorescence intensity of Lf‐LP‐DiR + Mu group was significantly lower than that of Lf‐LP‐Mu‐DiR group (*p* < 0.01) and somewhat higher than that of Lf‐LP‐DiR group. We speculated that muscone (gavage) had little effect on the lipophilicity of the targeted delivery system. However, muscone (gavage), as an aromatic resuscitation drug from Traditional Chinese Medicines, can promote Lf‐LP‐DiR to enter into the brain rapidly and increase Lf‐LP‐DiR elimination quickly, which may be due to the bidirectional permeation effect of muscone as previously reported.^4^

The result of in vivo anti‐glioma showed that the Lf‐LP‐Mu‐DTX group exhibited the best tumor suppressive effect, which was in agreement with the results of ex vivo imaging. The result suggested that Lf and muscone dual modified liposome had more favorable brain targeting ability, enhanced in vivo anti‐glioma effect, prolonged median survival time. In addition, the modified liposomes did not show systemic side effects.

In the process of the study, we found that some factors affected the efficacy of the targeted delivery system into the brain, including drug release, internalization, circulation, blood‐brain barrier recognition, and so forth.^53^ Changes in each process would affect the delivery efficiency of the brain‐targeting drug delivery system. Only by ensuring that the targeted drug delivery system with minimum loss in each process, it can enter the brain more and exert its curative effect. For example, modified nanoparticles can absorb plasma protein by way of specific and nonspecific interactions in systemic administration, which alters their physiochemical properties and following interactions with targeted cells.^54^

In traditional Chinese Medicines, aromatic resuscitation drugs were used to enhance the therapeutic effects of brain diseases and have a long history of application. In addition, the aromatic resuscitation drugs have good safety.^18^ In the present study, we used muscone as the “guide” drug and Lf as the target ligand to design brain targeting delivery system, in order to achieve improving brain targeting and therapeutic effect of glioma by Lf and muscone dual modification. Our study results showed that LF and muscone dual‐modified liposomes significantly enhanced brain targeting effect than by physical addition muscone group. Therefore, we think that muscone chemical modification is different from the physical addition of muscone on altering mechanisms of the BBB permeability. The further mechanisms for muscone chemical modification need to be explored in future studies. Our study would provide a reference for the brain targeting application of aromatic resuscitation drugs in traditional Chinese Medicines.

## 5 CONCLUSION

In conclusion, we established Lf and muscone dual‐modified DTX long‐circulating liposomes for glioma therapy, where Lf was modified for active targeting to LfR and muscone was modified for increasing the BBB permeability on the surface of the liposome. Lf and muscone dual‐targeted liposomes significantly enhanced the cellular uptake of glioma cells, in vivo brain targeting effect, and in vitro and in vivo anti‐glioma efficacy. The enhanced therapeutic efficacy of Lf‐LP‐Mu‐DTX may be explained that cationic Lf‐modified liposomes could increase cellular uptake by receptor‐ and adsorption‐mediated endocytosis pathways on the surface of glioma cells and cerebral vascular endothelial cells, and muscone modification could effectively penetrate across the BBB. Therefore, muscone modification could increase the brain targeting effect of macromolecule ligand Lf modified liposomes, Lf and muscone dual‐modified DTX long‐circulating liposomes enhanced the anti‐glioma efficacy in vitro and in vivo.

## AUTHOR CONTRIBUTIONS

**Wenjuan Duan:** Investigation (equal); methodology (lead); software (lead). **Duan Gao:** Investigation (equal); methodology (equal). **Ningzhu Ma:** Investigation (supporting); methodology (supporting). **Jianguo Zhang:** Investigation (supporting). **Jianfang Feng:** Conceptualization (equal).

## CONFLICT OF INTEREST

The authors declare no competing financial interest.

## Supporting information


**Appendix S1** Supporting Information.

## Data Availability

The data that supports the findings of this study are available in the supplementary material of this article.
