# Supplementary material for: “Guide” of muscone modification enhanced brain‐targeting efficacy and anti‐glioma effect of lactoferrin modified DTX liposomes
Source: Bioeng Transl Med. 2022 Aug 18;8(2):e10393. doi: 10.1002/btm2.10393 (PMC10013770; doi:10.1002/btm2.10393)
Supplement: Supplementary file 1 — Appendix S1 Supporting Information. [file BTM2-8-e10393-s001.docx]

**Supporting Information**

**“Guide” of muscone modification enhanced brain-targeting efficacy and anti-glioma effect of lactoferrin modified DTX** **liposomes**

*Na Qi ^a,b^，Wenjuan Duan^b,c^，Duan Gao^b^，Ningzhu Ma^b^， Jianguo Zhang^b^，Jianfang Feng ^d^，Aimin Li^a^**

a Cancer Center, Integrated Hospital of Traditional Chinese Medicine, Southern Medical University, Guangzhou, 510315, China;

b Department of Pharmacy, Guilin Medical University, Guilin, 541004, China;

c Department of Pharmacy, Affiliated hospital of Jinggangshan University, Ji’an, 343000, China;

d Department of Pharmacy, Guangxi University of Chinese Medicine, Nanning, 530299, China;

*Corresponding author at: Cancer Center, Integrated Hospital of Traditional Chinese Medicine, Southern Medical University, Guangzhou, 510315, China; E-mail address: liaimin2005@163.com (Aimin Li).

**Supporting Information Table and Figure of Contents Page**

**Table S1** Number of positive cells and Positive cells rate of liposome groups

on U87 cells (Mean±SD, n=3) 2

**Table S2** Number of positive cells and Positive cells rate of liposome groups

on hCMEC/D3 cells (Mean±SD, n=3) 3

Table S1 Number of positive cells and Positive cells rate of liposome groups on U87 cells (Mean±SD, n=3)

| Group | Number of positive cells | Positive cells rate (%) |
| --- | --- | --- |
| Blank | 9290±410 | 78.5±4.8 |
| PEG-LP-C6 | 8321±628 | 75.8±6.9 |
| LF-LP-C6 | 8990±587 | 78.6±1.3 |
| LF-LP-M-C6 | 9090±111 | 80.2±4.0 |
| LF-LP-C6+Mu | 8727±518 | 79.0±3.3 |

Table S2 Number of positive cells and Positive cells rate of liposome groups on hCMEC/D3 cells (Mean±SD, n=3)

| Group | Number of positive cells | Positive cells rate (%) |
| --- | --- | --- |
| Blank | 9382±500 | 79.4±3.9 |
| PEG-LP-C6 | 9763±116 | 77.0±2.0 |
| LF-LP-C6 | 9655±218 | 76.7±5.0 |
| LF-LP-M-C6 | 9139±92 | 70.9±1.8 |
| LF-LP-C6+Mu | 9775±247 | 76.0±4.2 |
